# Graphene Oxide Tablets for Sample Preparation of Drugs in Biological Fluids: Determination of Omeprazole in Human Saliva for Liquid Chromatography Tandem Mass Spectrometry

**DOI:** 10.3390/molecules24071191

**Published:** 2019-03-27

**Authors:** Zeynab Zohdi, Mahdi Hashemi, Abdusalam Uheida, Mohammad Mahdi Moein, Mohamed Abdel-Rehim

**Affiliations:** 1 Functional Materials Division, Department of Applied Physics, School of Engineering Sciences, KTH Royal Institute of Technology, Isafjordsgatan 22, Kista, SE-164 40 Stockholm, Sweden; zohdi_zeynab@yahoo.com (Z.Z.); salam@kth.se (A.U.); 2 Department of Clinical Neuroscience, Centre for Psychiatry Research, Karolinska Institutet, SE-171 76 Solna, Sweden; Mohammad.moein@ki.s; 3 Department of Chemistry, University of Bu-Ali Sina, Hamadan 65174, Iran; Mahdi.Hashemi@hotmail.com

**Keywords:** graphene oxide, omeprazole, liquid chromatography, tandem mass spectrometry, saliva, GO-Tabs

## Abstract

In this study, a novel sort of sample preparation sorbent was developed, by preparing thin layer graphene oxide tablets (GO-Tabs) utilizing a mixture of graphene oxide and polyethylene glycol on a polyethylene substrate. The GO-Tabs were used for extraction and concentration of omeprazole (OME) in human saliva samples. The determination of OME was carried out using liquid chromatography-tandem mass spectrometry (LC–MS/MS) under gradient LC conditions and in the positive ion mode (ESI+) with mass transitions of m/z 346.3→198.0 for OME and *m*/*z* 369.98→252.0 for the internal standard. Standard calibration for the saliva samples was in the range of 2.0–2000 nmol L^−1^. Limits of detection and quantification were 0.05 and 2.0 nmol L^−1^, respectively. Method validation showed good method accuracy and precision; the inter-day precision values ranged from 5.7 to 8.3 (%RSD), and the accuracy of determinations varied from −11.8% to 13.3% (% deviation from nominal values). The extraction recovery was 60%, and GO-Tabs could be re-used for more than ten extractions without deterioration in recovery. In this study, the determination of OME in real human saliva samples using GO-Tab extraction was validated.

## 1. Introduction

Omeprazole (OME) is a well-known drug that is used as a proton pump inhibitor to reduce the amount of acid produced in the stomach. OME is used in the treatment of all acid-related diseases, and it was introduced into the market for the first time by AstraZeneca under the brand name Losec^®^ [1]. The pure S-enantiomer of OME was subsequently commercialized under the name Nexium^®^. OME is classified as an effective and safe medicine [2]. Gas chromatography (GC) and liquid chromatography (LC) with mass spectrometric (MS) detection have been used for the determination of OME, and solid phase extraction (SPE) has often been utilized as a sample preparation technique for extracting OME from plasma samples [3,4].

Saliva provides a simple and non-invasive sample source compared to blood. Numerous publications report that saliva can be a good choice for the determination of drugs for diagnostic purposes. Several research groups have studied methods for determining (i.e., screening) different substances in oral fluid [5,6,7]. Until now OME was measured in saliva and plasma using HPLC [8] and liquid chromatography-tandem mass spectrometry (LC–MS/MS) [9] with simple extraction methods. However, developing a simple and novel sample preparation technique for measuring the OME in saliva samples is a demanding issue.

Sample preparation is the first and, oftentimes, the main step in bioanalytical methods. As a result, there is an increasing demand for biological sample preparation techniques that are simple, inexpensive and environmentally friendly, and that permit acceptable recovery and selectivity [10]. During the last two decades, several sample preparation techniques have been developed and directed towards automation and on-line coupling. Two of these techniques are microextraction by packed sorbent (MEPS) and molecularly imprinted polymer tablets (MIP-Tabs), both of which were developed and introduced by researchers in the Abdel-Rehim group. MEPS is a miniaturization form of SPE that reduces significantly the required amounts of sorbent, solvent and sample volume. The MEPS technique has been utilized to extract and enrich analytes from different matrices such as water, plasma, urine and blood [11,12,13,14,15]. MIP-Tabs were prepared using a thin film of MIP polymer on a polyethylene substrate as a support material, and have been applied to the extraction of methadone in plasma samples and amphetamine in urine samples with good recovery and precision [16,17].

Nano-materials are of interest in the field of sample preparation due to their unique properties—in this case, high surface area compared to bulk and microscale materials. High surface area provides high adsorption capacity and high pre-concentration factor [16]. Additionally, nano-materials can possess high chemical stability and can be easily functionalized to increase selectivity. In our recent work, reduced graphene oxide (GO) materials as a solid phase in the MEPS method, was successfully applied for extraction and measurement of local anesthesia in plasma and saliva samples [18]. GO as a potential candidate in biomedical application presents a large surface area and proper dispersibility in most solvents due to the formation of hydrogen bonds between polar functional groups of GO surface and water molecules [19]. Nowadays, there is still no promising available information about the in vitro and in vivo toxicity of GO, but it is well approved that the preparation of GO with high purity is a key factor from a safety aspect [20,21].

Here for the first time, a combination of graphene oxide and polyethylene glycol was used to prepare novel graphene oxide tablets (GO-Tabs) that were evaluated for the extraction of omeprazole in human saliva samples. GO-Tabs is a novel, straightforward and effective sample preparation trend which will be presented here for extraction of OME from saliva samples and measurement by LC–MS/MS.

## 2. Materials and methods

### 2.1. Chemicals and Reagents

OME (racemate) and (*S*)-Lansoprazole (internal standard, IS) were obtained from Sigma-Aldrich (Steinheim, Germany), and GO was obtained from Sigma-Aldrich (St. Louis, MO, USA). The polyethylene material (PE), with a pore size of 0.2 µm, was a commercial material and was obtained from Sigma-Aldrich and its surface was not chemically modified. This material is already used as filter for aqueous solutions. The tablet form was prepared by a homemade tool. HPLC grade acetonitrile and methanol were purchased from Merck (Darmstadt, Germany). Analytical grade formic acid and ammonium hydroxide were obtained from Merck (Darmstadt, Germany). A Milli-Q Plus water purification system from Millipore (Bedford, MA, USA) was used for water purification.

### 2.2. Instrumentation

The LC system used in this study included two Shimadzu pumps (LC-10ADvp, Kyoto, Japan) and an autosampler (CTC-Pal, Analytics AG, Zwingen, Switzerland) with 50 µL sample loop. The separation column was 100 mm × 2.1 mm i.d. packed with 3.5 μm Zorbax Bonus-RP particles (Agilent, Palo Alto, CA, USA). The LC mobile phase A was 0.1% formic acid in acetonitrile/water (0.5:99.5 *v*/*v*) and mobile phase B contained 0.1% formic acid in acetonitrile/water (80:20 *v*/*v*). The mobile phase gradient was 30% to 90% phase B in 5 min, with a final hold at 90% B for 2 min before resetting at 30% phase B. The mobile phase flow rate was held constant at 0.6 mL min^−1^.

The MS instrumentation consisted of a triple quadrupole MS analyzer (Quatro-micro, Waters, Manchester, UK) equipped with an electrospray ionization source (ESI+) operated in the positive ion mode. The MS source block and desolvation temperatures were set at 150 °C and 350 °C, respectively. Nitrogen was utilized as curtain gas (950 L h^−1^), and argon was utilized as the collision gas (collision energy of 10 eV for OME and 20 eV for lansoprazole). OME analysis in saliva samples was performed by using multiple reaction monitoring (MRM) transitions of *m*/*z* 346.0 > 198.0 for OME and *m*/*z* 369.9 > 252.0 for the IS with a dwell time of 0.2 s/transition. Peak-area ratios (OME/IS) were used for all calculations. Data analysis was performed using MassLynx software (version 4.1) obtained from Waters (Manchester, UK).

### 2.3. Preparation and Characterization of GO-Tabs

**Figure 1:**
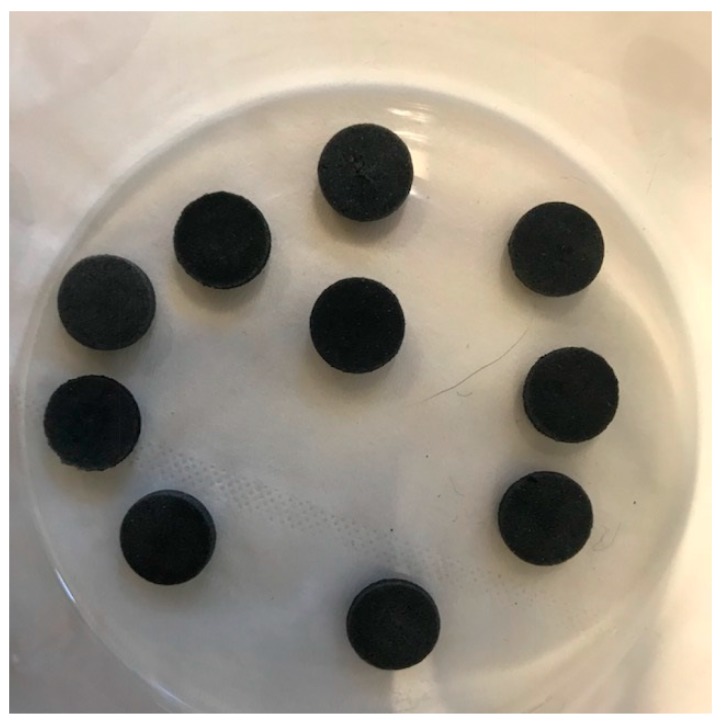
Photograph of prepared graphene tablets (GO-Tabs).

Polyethylene substrates in the tablet form (9 mm diameter × 2 mm thickness) were washed with HCL (1 M) and then NaOH (1 M) in an ultrasonic bath for 10 min, and then they were washed with water and dried at room temperature. Graphene oxide (20 mg) was added to 10 mL of acetonitrile and ultrasonicated for 30 min, and then 25 mg of polyethylene glycol (PEG) was added to the graphene oxide suspension. PEG was used to improve the interfacial adhesion between the GO nano-particles and the polyethylene tablet surface on which they were coated, and the dispersion of GO in the resultant film. Blank polyethylene tablets (10 total) were immersed in the GO-PEG suspension and ultrasonicated for 1–4 h; it was found that 3 h was the optimum time. After ultrasonication, the tablets were removed and placed in a freeze dryer overnight. Figure 1 shows a photograph of some prepared GO-Tabs. The GO-Tabs were chemically and mechanically stable.

### 2.4. Preparation of Stock, Standard and Quality Control Solutions

Two stock solutions (100 µM each) were prepared in methanol [one for preparing standard samples and the other for preparing quality control (QC) samples]. The standard and QC samples were prepared in blank pooled human saliva samples (*n* = 6). The concentrations of standard compounds in the saliva were in the range of 2.0–2000 nM. The QC samples were prepared in saliva at three concentration levels: Low (QCL, 6 nM), medium (QCM, 900 nM), and high (QCH, 1600 nM). 

### 2.5. Sample Preparation

Standard samples in saliva were freshly prepared for each validation assay, while QC samples in saliva were prepared and stored at −20 °C until needed. A 200 μL volume of each sample was mixed with a 100 μL of the IS (1000 nM in methanol), then diluted with water (1:4) and centrifuged for 3 min. Then a GO-Tab was immersed in each saliva sample and shaken for 10 min, after which it was removed and washed with 200 μL of water. Then the analyte and internal standard were desorbed (i.e., extracted) by soaking in 1.0 mL of methanol for 1.0 min. The eluates were evaporated to dryness and redissolved in 200 μL of LC mobile phase. A 30 μL volume of the final sample solution was injected into the LC–MS/MS for analysis.

## 3. Results and Discussion

In this study, GO-Tabs were prepared and investigated for the extraction of OME in human saliva samples. Factors affecting the extraction performance, including desorption solution, extraction time, desorption time, sample pH, sample concentration and adsorption capacity, were investigated to obtain the best extraction/recovery efficiency.

### 3.1. GO-Tab Morphology Analysis

**Figure 2:**
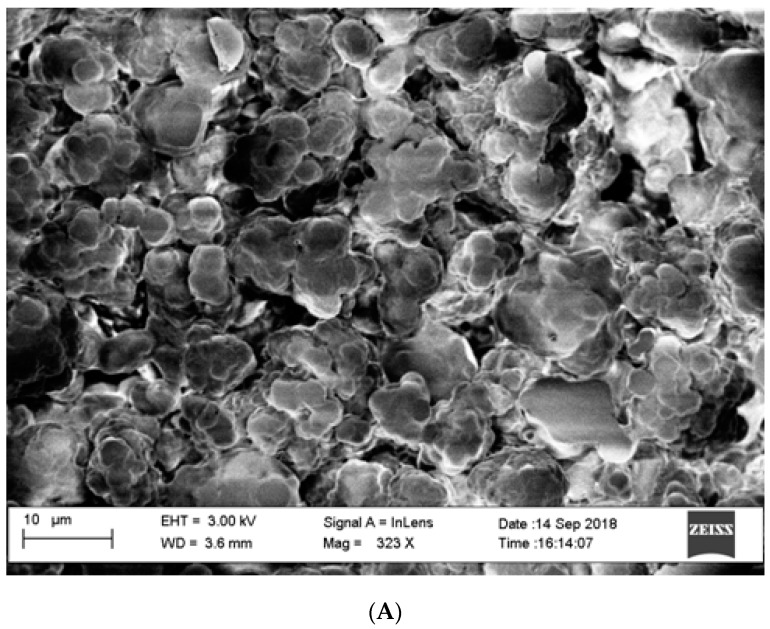

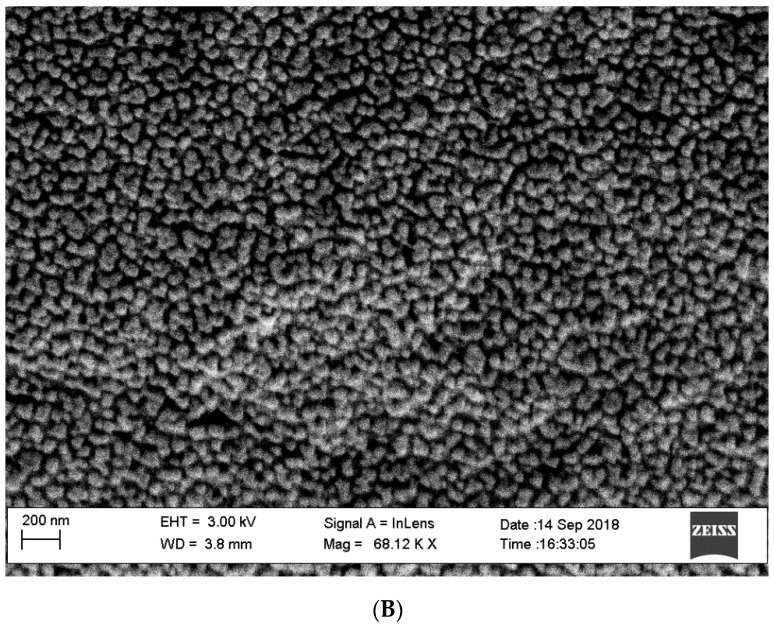
Electron micrographs (SEM) of GO-Tabs (**A**) before and (**B**) after polymerization.

As described above, thin layers of GO-PEG were absorbed into the pores and surface of a polyethylene film and frozen overnight. The resultant GO-Tabs were 9 mm in diameter and 2 mm in thickness (Figure 1). SEM images before (Figure 2A) and after (Figure 2B) GO-PEG addition clearly show pores (Figure 2A) that become covered with the GO-containing polyethylene film (Figure 2B).

### 3.2. Optimization of Extraction Protocol

#### 3.2.1. Extraction Time

**Figure 3:**
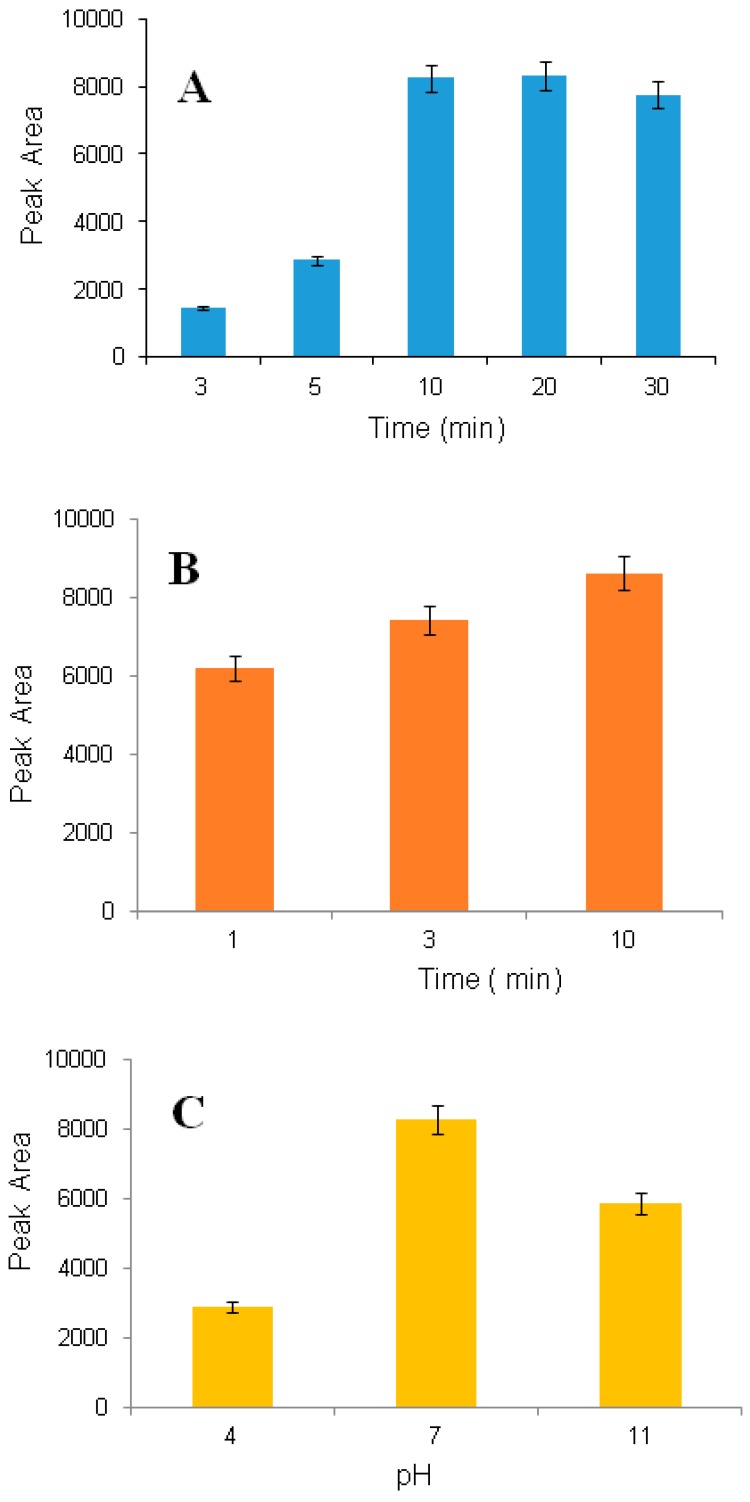
Effect of extraction time on extraction recovery (saliva sample, 1600 nmol L^−1^) (**A**), effect of desorption time on extraction recovery (saliva sample, 1600 nmol L^−1^) (**B**) and effect of sample pH (saliva sample, 1600 nmol L^−1^) (**C**); pH was adjusted by addition of ammonium hydroxide and formic acid.

Because mass transfer is a time dependent process, the extraction time is an important factor. The effect of time on extraction recovery was investigated for 3.0, 5.0, 10.0, 20.0 and 30.0 min. The recovery was increased significantly when the time increased from 3.0 to 10.0 min (Figure 3A).

#### 3.2.2. Desorption Time

The effect of desorption time on extraction efficiency was investigated for 1.0, 3.0 and 10.0 min, and the best result was obtained with 10.0 min (Figure 3B). After 10.0 min, no significant improvement was observed.

#### 3.2.3. Type of Desorption Solvent

Desorption of extracted analyte from GO-Tabs was studied utilizing different solvents, including methanol, mixtures of methanol and water, and acetonitrile. Acetonitrile gave the highest recovery.

#### 3.2.4. Effect of pH

The effect of sample pH on extraction recovery was investigated for three different pH values: Low (3), neutral (7) and high (12). The highest extraction yield was observed at neutral pH (Figure 3C).

### 3.3. Adsorption Capacity

**Figure 4:**
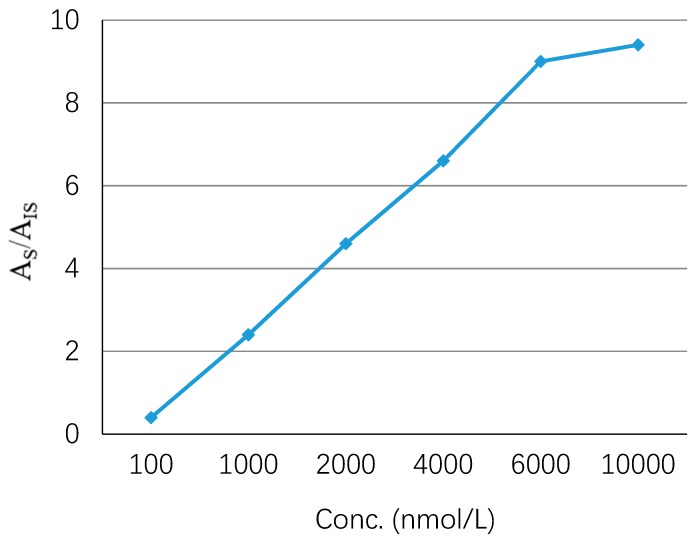
Capacity of GO-Tabs.

The adsorption capacity indicates the ability of the GO-Tabs to adsorb a specific analyte. In order to study the adsorption capacity of the GO-Tabs for OME, a series of different concentrations of OME in saliva samples ranging from 0.1 to 10 µmol L^−1^ were prepared. The extraction recovery was linear up to 6.0 µmol L^−1^, and then the GO-Tab became saturated as shown in Figure 4.

### 3.4. Selectivity of the GO-Tabs

**Figure 5:**
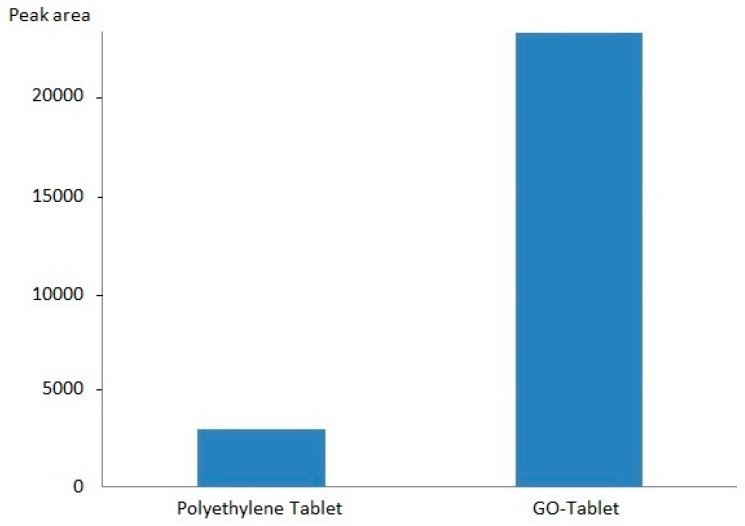
Efficiency of GO-Tabs and polyethylene blank tablet (saliva sample, 2000 nmol L^−1^).

The GO-Tab selectivity was investigated by comparing the extraction of OME in saliva using GO-Tabs and bare polyethylene tablets. The extraction recovery (recorded as area) using GO-Tabs was 4-fold higher compared with uncoated polyethylene tablets (Figure 5).

### 3.5. Method Validation

Validation of the method described in this study for determination of OME in human saliva was carried out according to international guidelines [22,23] and included linearity, limit of quantification (LLOQ), accuracy, precision, recovery, matrix effects, selectivity and carry-over.

#### 3.5.1. Calibration, Selectivity and Extraction Efficiency

A standard calibration curve was constructed using eight OME standards prepared in saliva over the concentration range from 2.0 to 2000 nmol L^−1^. A quadratic regression equation with 1/x weighting was used. The coefficient of determination (R^2^) was 0.99 or higher for all analyses (*n* = 3). The limit of detection (LOD) was found to be 0.1 nmol L^−1^ and the limit of quantification (LLOQ) was equal to the lowest standard concentration (2.0 nmol L^−1^) used to construct the calibration plot.

To evaluate the method selectivity, six different saliva samples were used. A saliva blank without OME and internal standard was analyzed and compared with a chromatogram obtained using a sample with OME concentration at the limit of quantification (LLOQ) to confirm the absence of endogenous interfering peaks at the same retention time of the OME analyte in the chromatograms. No significant peaks (≥20% of the LLOQ) were observed at the same retention time as OME and the I.S. The method extraction recovery was found to be between 80 to 90%.

#### 3.5.2. Accuracy and Precision

The relative error method (i.e., percent difference between the determined mean concentrations and the true concentrations) was used to evaluate accuracy, and the precision was calculated as the percentage of the relative standard deviation for the analysis of the quality control samples. For validation, three assays were done, and each assay consisted of eight calibration points and six quality control (QC) sample replicates at three concentration levels: Low (QCL: 6 nmol L^−1^), medium (QCM: 900 nmol L^−1^), and high (QCH: 1600 nmol L^−1^). The accuracy was found to be in the range of 88.0–106.0% (*n* = 18), and intra- and inter-assay precisions were found to be in the range of 4.1–5.4% (*n* = 6) and 5.6–7.8% (*n* = 18), respectively (Table 1).

#### 3.5.3. Method Selectivity and Matrix Effects

**Figure 6:**
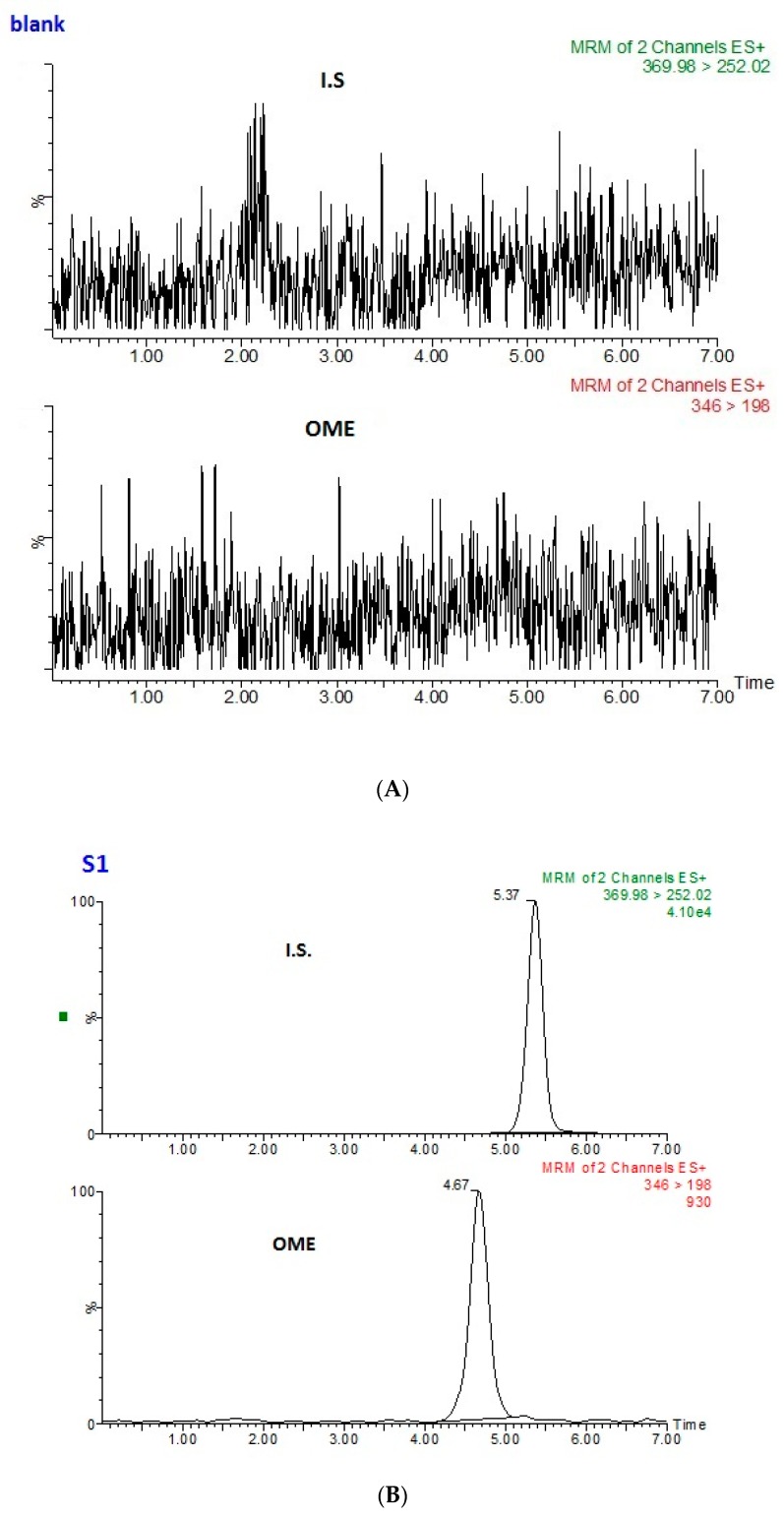
(**A**) Multiple reaction monitoring (MRM) transitions obtained from the analysis of a blank saliva sample, and (**B**) OME at 2 nmol L^−1^ (S1) with internal standard.

The method selectivity was examined by comparing LC–MS/MS chromatograms of the blank saliva sample (Figure 6A) and a standard sample spiked with internal standard and OME (Figure 6B). The blank saliva did not introduce any interfering peaks near the retention time of OME and the internal standard.

The effect of the saliva matrix on the MS signal was evaluated using the post-extraction addition method. Blank saliva was extracted according to the described protocol and OME was added to the extract at two concentration levels (QCL and QCH). Comparing LC–MS/MS analyses of these and pure methanol samples containing the same concentrations of OME showed that the saliva matrix did not affect the detector signal to any noticeable extent. Matrix effects ranged from –3% (using LQC) to −1% (using HQC).

#### 3.5.4. Carry-Over and Reuse of GO-Tabs

After each extraction, the GO-Tab was washed first with methanol and then with water to eliminate carry-over into the next extraction. No carry-over could be detected when a blank saliva sample was extracted immediately after extraction of the highest concentration standard. A single GO-Tab could be re-used for ten extractions without any observable change in extraction efficiency (*n* = 10, SD = 2.4 and %RSD = 4.0).

## 4. Analysis of Patient Samples

**Figure 7:**
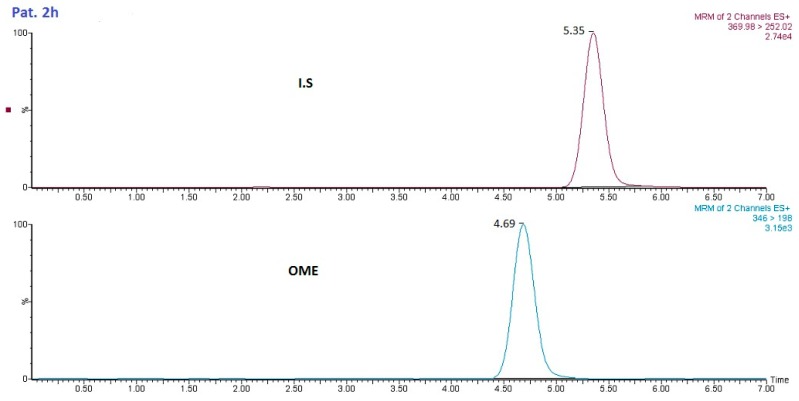
Chromatogram of OME from a patient sample (2 h after administration).

The methodology developed in this study was used for the analysis of saliva samples from healthy subjects after administration of OME (20 mg dosage). Saliva samples were collected and analyzed for OME. Figure 7 shows the LC–MS/MS analysis of a patient saliva sample 2 h after administration of 20 mg of OME.

## 5. Conclusions

In this study, GO-Tabs were prepared using a novel sample preparation sorbent. GO was mixed with polyethylene glycol in acetonitrile and absorbed into a film of polyethylene using an ultrasonic bath. Polyethylene glycol was used to improve the dispersion of GO in the polyethylene substrate and the interfacial adhesion between GO and the substrate. This resulted in the formation of a layer of GO on the surface and within the pores of the polyethylene scaffold (Figure 1 and Figure 2). Validation experiments demonstrated that this method accurately determined OME in human saliva samples with high precision and good sensitivity. The GO-Tabs could be re-used for at least up to ten extractions. GO-Tabs are a novel advance in sample preparation sorbent development and as a potential sorbent, it can be applied in other complex solutions in the near future.

## Figures and Tables

**Table 1:** Precision of quality control (QC) samples of omeprazole (OME) in human saliva.

Compound	Sample (Conc.)	Accuracy (%) (*n* = 18)	Precision (RSD%)	
			Intra-day (*n* = 6)	Inter-day (*n* = 18)
Omeprazole	QCL (6.0 nM)	105.5	5.4	7.8
	QCM (800 nM)	106.4	5.1	7.5
	QCH (1600 nM)	87.8	4.1	5.6
